# Association between gut microbiota and glioblastoma: a Mendelian randomization study

**DOI:** 10.3389/fgene.2023.1308263

**Published:** 2024-01-04

**Authors:** Song Wang, Fangxu Yin, Zheng Guo, Rui Li, Wei Sun, Yuchao Wang, Yichen Geng, Chao Sun, Daqing Sun

**Affiliations:** ^1^ Department of Pediatric Surgery, Tianjin Medical University General Hospital, Tianjin, China; ^2^ Nursing College of Binzhou Medical University, Yantai, Shandong, China; ^3^ Department of Orthopedic Surgery, Tianjin Medical University General Hospital, Tianjin, China

**Keywords:** glioblastoma, gut microbiota, gut-brain axis, Mendelian randomization, causality

## Abstract

**Background:** Glioblastoma (GBM) is the most prevalent malignant brain tumor, significantly impacting the physical and mental wellbeing of patients. Several studies have demonstrated a close association between gut microbiota and the development of GBM. In this investigation, Mendelian randomization (MR) was employed to rigorously evaluate the potential causal relationship between gut microbiota and GBM.

**Methods:** We utilized summary statistics derived from genome-wide association studies (GWAS) encompassing 211 gut microbiota and GBM. The causal association between gut microbiota and GBM was scrutinized using Inverse Variance Weighted (IVW), MR-Egger, and Weighted Median (WM) methods. Cochrane’s Q statistic was employed to conduct a heterogeneity test. MR-Pleiotropic Residuals and Outliers (MR-PRESSO) were applied to identify and eliminate SNPs with horizontal pleiotropic outliers. Additionally, Reverse MR was employed to assess the causal relationship between GBM and pertinent gut microbiota.

**Results:** The MR study estimates suggest that the nine gut microbiota remain stable, considering heterogeneity and sensitivity methods. Among these, the *family.Peptostreptococcaceae* and *genus.Eubacterium brachy group* were associated with an increased risk of GBM, whereas *family.Ruminococcaceae*, *genus.Anaerostipes*, *genus.Faecalibacterium*, *genus.LachnospiraceaeUCG004*, *genus.Phascolarctobacterium*, *genus.Prevotella7*, and *genus.Streptococcus* were associated with a reduced risk of GBM. Following Benjamini and Hochberg (BH) correction, *family.Ruminococcaceae* (OR = 0.04, 95% CI: 0.01–0.19, FDR = 0.003) was identified as playing a protective role against GBM.

**Conclusion:** This groundbreaking study is the first to demonstrate that *family.Ruminococcaceae* is significantly associated with a reduced risk of GBM. The modulation of *family_Ruminococcaceae* for the treatment of GBM holds considerable potential clinical significance.

## 1 Introduction

Glioblastoma (GBM) is one of the most prevalent types of malignant brain tumors, with an annual incidence ranging from 3 to 6.4 per 100,000 individuals. It constitutes approximately 23.3% of central nervous system tumors and 78.3% of malignant brain tumors. The 5-year mortality rate ranks second only to that of pancreatic cancer and lung cancer (Sung et al., 2021; Ostrom et al., 2023). Typically arising from glial cells or precursor cells, its clinical manifestations encompass increased intracranial pressure, neurological and cognitive impairment, as well as seizures (Omuro and DeAngelis, 2013). According to the World Health Organization (WHO) classification, gliomas are categorized into four grades, with a direct correlation between higher grade and poorer prognosis. Notably, GBM stands out as the most malignant subtype. Characterized by a suppressive immune microenvironment and a grim prognosis, GBM stands as one of the most challenging tumors, prone to recurrence and imposing a substantial societal burden (Chen et al., 2021).

There is mounting evidence that the immunosuppressive environment of GBM is not only mediated by the immunosuppressive cells and molecules described above but also has many connections to the gut microbiota that contribute to the development of GBM (5). The human gut microbiota contains microbes with diverse properties and functions. Imbalance in the gut microbiota refers to the inability of bacteria in the human environment to maintain a dynamic balance, resulting in an imbalance of gut microbiota. Bacteria in the human environment are unable to maintain homeostasis, leading to inflammation and immunosuppression, and the gut microbiota is particularly responsive to the presence of tumors (Ferreiro et al., 2018; Sepich-Poore et al., 2021). In recent years, the role of the gut microbiota in tumors has been extensively studied. In neurodegenerative diseases and tumors of the central nervous system (CNS), the gut microbiota establishes interactions between the gut and the CNS in complex and as yet unclear ways (Fung et al., 2017).

Given the ethical issues and costs associated with clinical trials, determining causation becomes challenging (Bothwell and Podolsky, 2016). Many studies investigating the relationship between the gut microbiota and tumors have primarily employed case-control designs, introducing difficulty in establishing the temporal sequence between changes in the composition of the gut microbiota and the onset of tumors (de Clercq et al., 2021; Bellerba et al., 2022; Reichard et al., 2022). In light of these challenges, Mendelian randomization (MR) emerges as a robust approach, utilizing single nucleotide polymorphisms (SNPs) as instrumental variables (IV) derived from genome-wide association studies (GWAS) to ascertain causality between exposure and outcome (Sekula et al., 2016). Consequently, our present study employs Mendelian randomization methods to analyze the causal association between gut microbiota and glioblastoma multiforme (GBM), providing insights for potential clinical interventions for GBM.

## 2 Materials and methods

### 2.1 Study population

**FIGURE 1:**
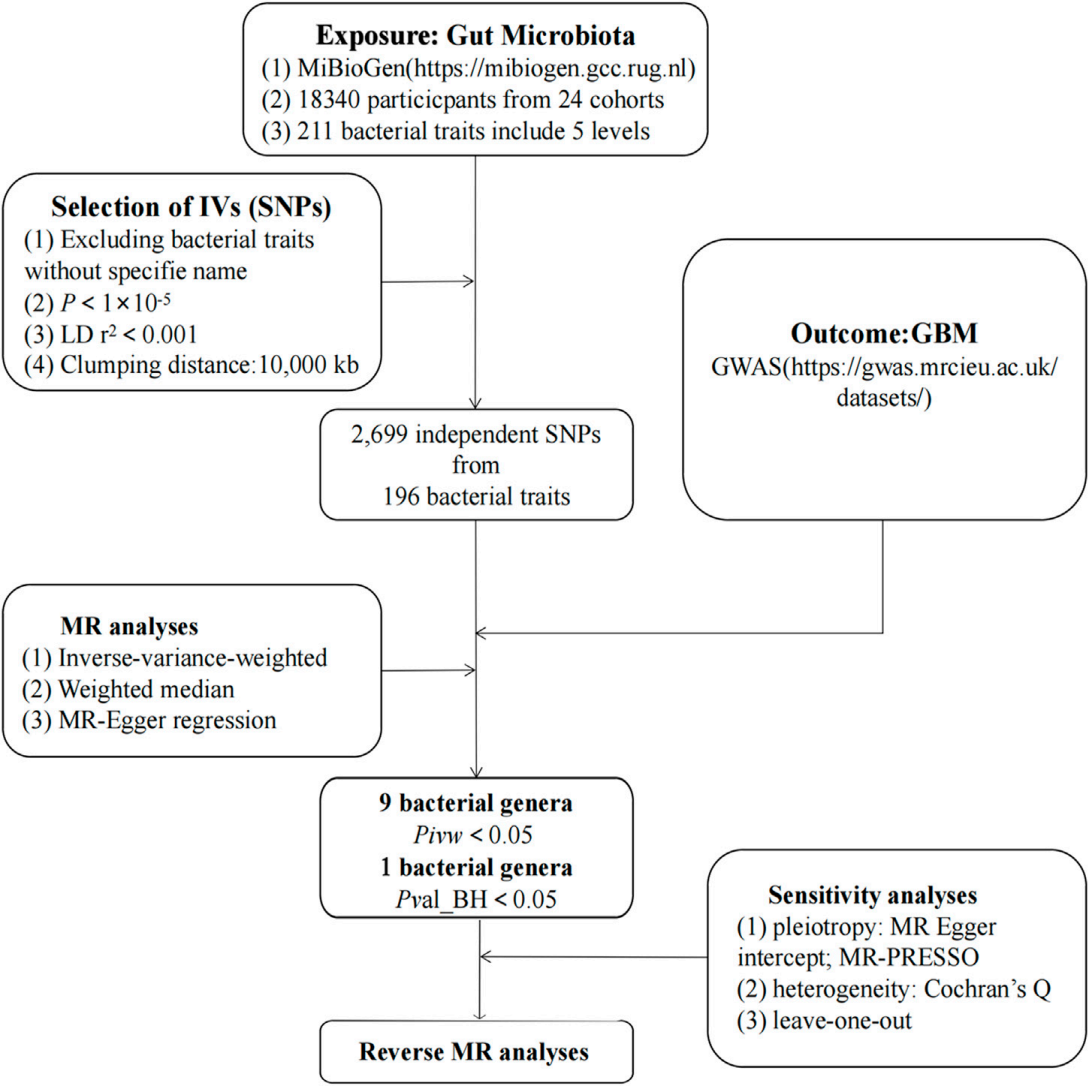
The study design of the present MR study of the associations of gut microbiota and GBM. Abbreviations: GBM, glioblastoma; LD, linkage disequilibrium, which used to measure the correlations between SNPs; IVW, Inverse Variance Weighted, the main analyses to evaluate the relationship between exposure and outcome; MR-PRESSO, Mendelian Randomization Pleiotropy RESidual Sum and Outlier, a method test the pleiotropic biases in the SNPs and correct the pleiotropic effects; MR, Mendelian randomization; SNP, single nucleotide polymorphism, as instrumental variables for the exposures and outcomes.

**FIGURE 2:**
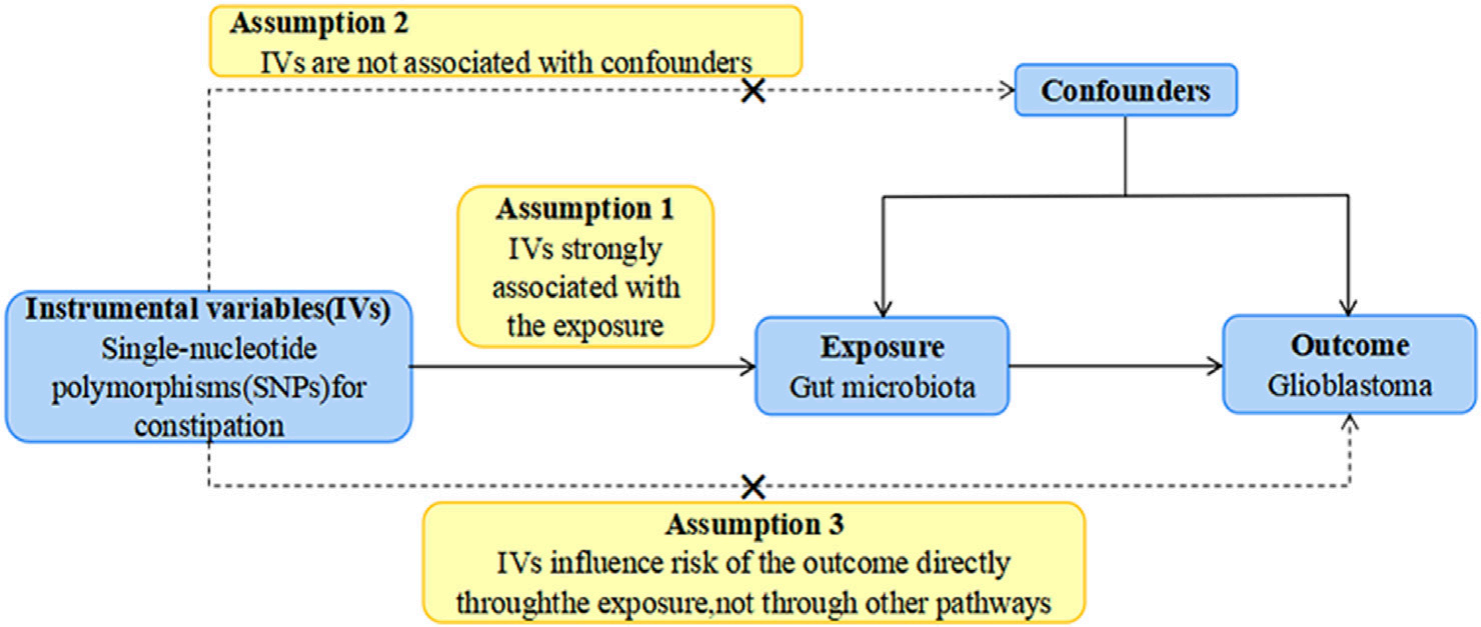
The study design of the present MR study of the associations of gut microbiota and GBM. Abbreviations: GBM, glioblastoma; LD, linkage disequilibrium, which used to measure the correlations between SNPs; IVW, Inverse Variance Weighted, the main analyses to evaluate the relationship between exposure and outcome; MR-PRESSO, Mendelian Randomization Pleiotropy RESidual Sum and Outlier, a method test the pleiotropic biases in the SNPs and correct the pleiotropic effects; MR, Mendelian randomization; SNP, single nucleotide polymorphism, as instrumental variables for the exposures and outcomes.

As illustrated in Figure 1, our study outlines the two-sample MR investigation employed to explore the causal association between the gut microbiota and GBM. Subsequently, rigorous quality controls, including heterogeneity and gene pleiotropy tests, were executed to validate the dependability of the causal findings. In enhancing the precision of causal effect estimation, adherence to three crucial assumptions is imperative when utilizing SNPs as IVs in MR analysis (Sung et al., 2021): IVs must be closely aligned with the exposure factor; (Ostrom et al., 2023) IVs should exhibit no correlation with confounding factors; (Omuro and DeAngelis, 2013); IVs must exclusively influence outcomes through exposure, avoiding other pathways (Figure 2).

The main exposure factor in our study is the gut microbiota, and we investigate human genetics within the context of studying the gut microbiota. This investigation is conducted as part of an international consortium known as MiBioGen (Kurilshikov et al., 2021). Our study encompasses data from the human gut microbiota of 18,340 European individuals derived from 24 population-based cohorts. After adjustment for age, sex, technical covariates, and genetic principal components, spearman’s correlation analysis was performed to identify genetic loci that affected the covariate-adjusted abundance of bacterial taxa. Following the exclusion of 15 genera lacking specific species names, we identified 196 bacterial taxa, comprising 9 phyla, 16 orders, 20 orders, 32 families, and 119 genera.

The outcome variable we focus on is GBM, and the GWAS dataset associated with GBM came from a publicly available GWAS meta-analysis that included 91 cases and 218,701 controls of European ancestry (Sudlow et al., 2015). The GWAS meta-analysis, a prospective cohort study, systematically gathers comprehensive genetic and phenotypic data from approximately 500,000 individuals across the UK. Each participant contributes a wealth of phenotypic and health-related information. Genome-wide genotype data were collected for all participants by linking health and medical records to provide comprehensive follow-up information.

### 2.2 Selection of instrumental variables

Single nucleotide polymorphisms (SNPs) are the most frequently utilized genetic variations in MR Analysis, which mainly refers to the DNA sequence diversity caused by a change in a single nucleotide at the genomic level. In this study, SNPs significantly associated with the relative abundance of 196 gut microbiota were selected as the available instrumental variables (IVs). Previous studies have shown that the inclusion of multiple instrumental variables enhances the explanatory power of the observed variation and enhances the accuracy and reliability of the analyzed results. Therefore, in this study, the selection of IVs was based on the results of correlation analysis where significance was determined at P < 1 × 10^−5^. The criteria for linkage disequilibrium were set at R^2^ < 0.001 and a genetic distance of 10,000 kb, whereby highly correlated SNPs were excluded to ensure the independence of the included SNPs from each other. Finally, SNPs associated with the relative abundance of gut microbiota were projected to the GWAS pooled data of GBM, and the corresponding statistical parameters were extracted. Utilizing statistical parameters associated with identical loci in the relative abundance of gut microbiota and the GWAS results for GBM, the data were harmonized. This harmonization ensured that the effect values for both exposure and outcome corresponded to the same effect allele.

### 2.3 Statistical analysis

In this study, Inverse Variance Weighted (IVW), MR-Egger, Weighted Median (WME) were used to estimate the dependent effects. The IVW method operates under the assumption that all genetic variants are valid IVs. It employs the ratio method to calculate the causal effect values for individual instrumental variables, subsequently summarizing each estimate through a weighted linear regression to derive the total effect value. Notably, the main divergence between the MR-Egger method and the IVW method lies in the regression, which takes into account the presence of an intercept term. Conversely, the WME method strategically leverages the intermediate effects of all available genetic variants, obtaining estimates by weighting the inverse variance of the correlation of each SNP with the outcome.

Since the IVW method exhibits higher test efficacy compared to other MR methods, we chose it as the preferred method for estimating causal effects in this study. Additionally, for enhanced result interpretation, the study transformed Beta (β) values obtained from the results into Odds Ratios (OR), while simultaneously calculating the 95% confidence intervals (CI). To assess the association of effect estimates for causality, which might be influenced by weak instrumental bias, the strength of IV was evaluated using the F statistic. This statistic was calculated using the following equation: F = R^2^ (n-k-1)/k (1-R^2^), where R^2^ represents the variance explained by IV (for each gut microbiota), and n is the sample size. The value of R^2^ was estimated using the minor allele frequency (MAF), and b values were determined by the equation: R2 = 2 × MAF × (1-MAF) × b^2^.

In addition, for the purpose of further testing the stability and reliability of the results, quality control included sensitivity analysis and heterogeneity testing, as well as a gene multiplicity test. Sensitivity analysis was performed using the leave-one-out method, where the combined effect values of the remaining SNPs were calculated by sequentially deleting individual SNPs, and the effect of each SNP on the results was assessed. Heterogeneity testing was conducted using the Cochran Q test to determine the heterogeneity of the SNPs, aiming to assess the possible bias in the estimation of the causal effect due to the measurement error of SNPs caused by different analysis platforms, experimental conditions, and analyzing populations. Horizontal gene pleiotropy tests were employed to assess whether IVs affected outcomes through pathways other than exposure, utilizing intercept terms from MR-Egger regression. Finally, reverse MR was performed to analyze whether there was a reverse causal relationship between GBM and meaningful gut microbiota. MR analyses and quality control for this study were conducted using version 4.0.3 of R and additionally version 0.5.6 of the TwoSampleMR software package.

## 3 Results

### 3.1 Two-sample Mendelian randomization

The results of this study involving gut microbiota associated with GBM are presented in Supplementary Table S1. After a series of quality control steps, 136 independent SNPs from 9 gut microbiota were associated with GBM. The F-statistics for the gut microbiota ranged from 14.58 to 88.42, and all met the threshold of greater than 10, suggesting that they are unlikely to be affected by weak instrumental bias (Supplementary Table S2). Briefly, we identified nine gut microbiota associated with GBM. After undergoing BH correction, the *family.Ruminococcaceae* was found to play a protective role against GBM (Table 1). Details of the IVs used are listed in Supplementary Table S3.

**TABLE 1:** Effect estimation of the association between meaningful gut microbiota and risk of GBM in MR analysis. Abbreviations: GBM, glioblastoma; MR, Mendelian randomization analysis; SNPs, Number of single nucleotide polymorphism. CI, confidence interval; OR, odds ratio; *P*_FDR_, *p*-value was calculated by the Benjamini-Hochberg method.

Gut microbiota	Outcome	SNPs	Methods	OR (95% CI)	*p*-value	*P*_FDR_
***family.Peptostreptococcaceae***	**GBM**					
		13	MR-Egger	2.34 (0.10–52.17)	0.603	
		13	Weighted median	6.42 (1.09–37.71)	0.040	
		13	IVW	3.83 (1.02–14.35)	0.046	0.566
***family.Ruminococcaceae***	**GBM**					
		9	MR-Egger	0.02 (3.89E-4-0.72)	0.070	
		9	Weighted median	0.08 (0.01–0.79)	0.031	
		9	IVW	0.04 (0.01–0.19)	9.51E-5	0.003
***genus.Anaerostipes***	**GBM**					
		13	MR-Egger	2.94 (0.01–1058.94)	0.727	
		13	Weighted median	0.34 (0.04–2.78)	0.312	
		13	IVW	0.16 (0.03–0.83)	0.029	0.680
***genus.Eubacterium brachy group***	**GBM**					
		10	MR-Egger	0.96 (0.03–36.27)	0.984	
		10	Weighted median	4.70 (1.46–15.14)	0.009	
		10	IVW	2.85 (1.16–7.01)	0.023	0.680
***genus.Faecalibacterium***	**GBM**					
		18	MR-Egger	0.31 (0.02–4.96)	0.434	
		18	Weighted median	0.18 (0.02–1.52)	0.115	
		18	IVW	0.16 (0.04–0.65)	0.011	0.632
***genus.Lachnospiraceae UCG004***	**GBM**					
		24	MR-Egger	0.09 (1.42E-4-61.80)	0.491	
		24	Weighted median	0.32 (0.04–2.53)	0.281	
		24	IVW	0.20 (0.04–0.96)	0.045	0.758
***genus.Phascolarctobacterium***	**GBM**					
		12	MR-Egger	0.03 (1.20E-5-75.19)	0.414	
		12	Weighted median	0.65 (0.08–5.60)	0.694	
		12	IVW	0.19 (0.04–0.93)	0.041	0.680
***genus.Prevotella7***	**GBM**					
		27	MR-Egger	0.82 (0.01–92.06)	0.936	
		27	Weighted median	0.28 (0.10–0.84)	0.023	
		27	IVW	0.30 (0.13–0.68)	0.004	0.458
***genus.Streptococcus***	**GBM**					
		10	MR-Egger	0.02 (6.59E-5- 3.57)	0.159	
		10	Weighted median	0.27 (0.03–2.16)	0.215	
		10	IVW	0.21 (0.05–0.97)	0.046	0.758

### 3.2 Causal effects of gut microbiota on GBM

**FIGURE 3:**
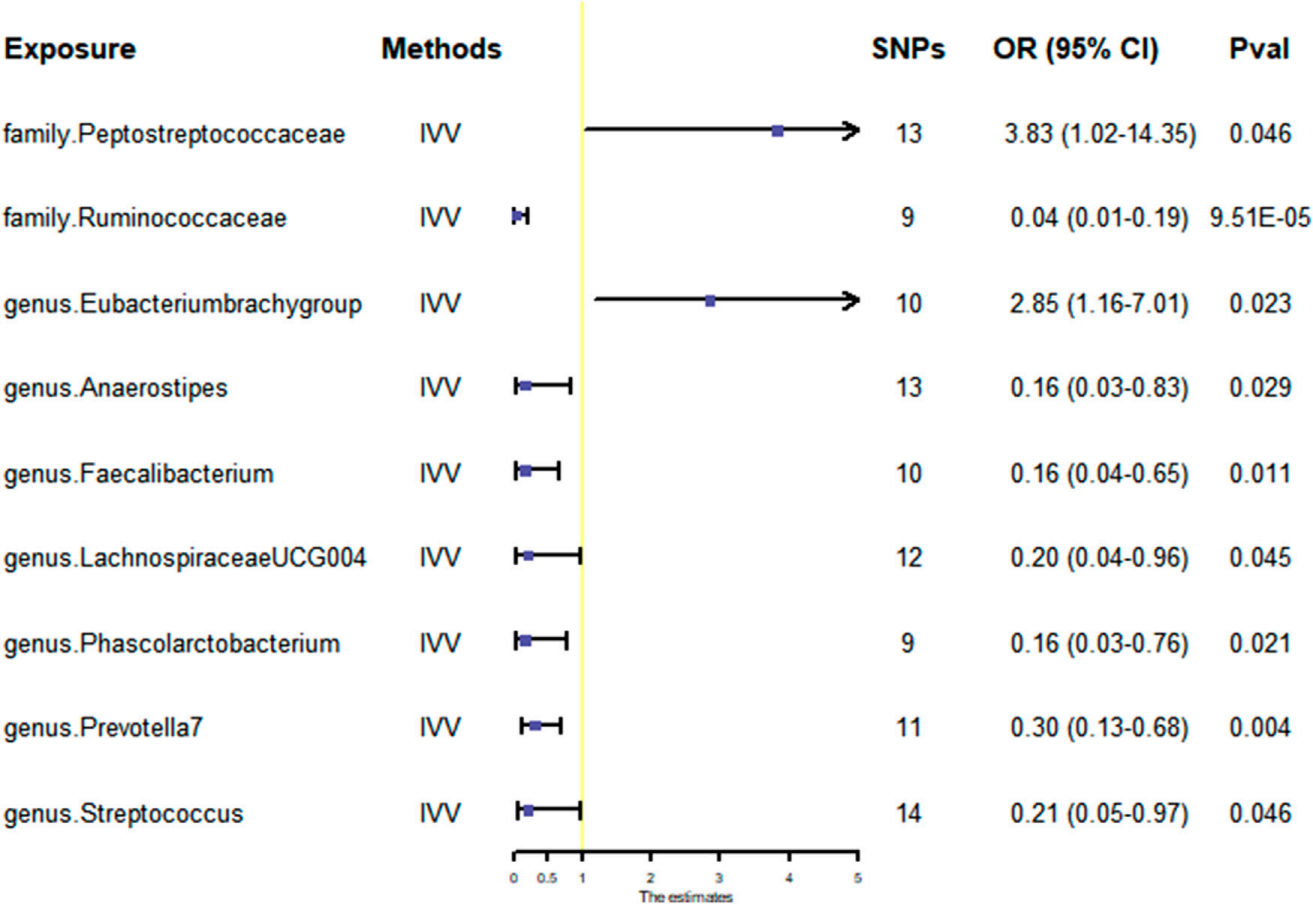
Scatter plots for the causal association between gut microbiota and GBM. Abbreviations: GBM, glioblastoma; OR, odds ratio; CI, confidence interval.

Nine gut microbiota were screened for correlation with GBM according to the IVW (Figure 3). Among them, *family.Peptostreptococcaceae* (OR: 3.83, 95% CI: 1.02–14.35, *p* = 0.046) and *genus.Eubacterium brachy group* (OR: 2.85, 95% CI: 1.16–7.01, *p* = 0.023) were found to increase the risk of GBM, while *family.Ruminococcaceae* (OR: 0.04, 95% CI: 0.01–0.19, *p* = 9.51E-05), *genus.Anaerostipes* (OR: 0.16, 95% CI: 0.03–0.83, *p* = 0.029), *genus.Faecalibacterium* (OR: 0.16, 95% CI: 0.04–0.65, *p* = 0.011), *genus.Lachnospiraceae UCG004* (OR: 0.20, 95% CI: 0.04–0.96, *p* = 0.045), *genus.Phascolarctobacterium* (OR: 0.16, 95% CI: 0.03–0.76, *p* = 0.021), *genus.Prevotella7* (OR: 0.30, 95% CI: 0.13–0.68, *p* = 0.004), and *genus.Streptococcus* (OR: 0.21, 95% CI:0.05–0.97, *p* = 0.046) showed a negative correlation with GBM. However, only *family.Ruminococcaceae* was found to be negatively associated with the risk of GBM after strict BH correction (P_FDR_ = 0.003).

**FIGURE 4:**
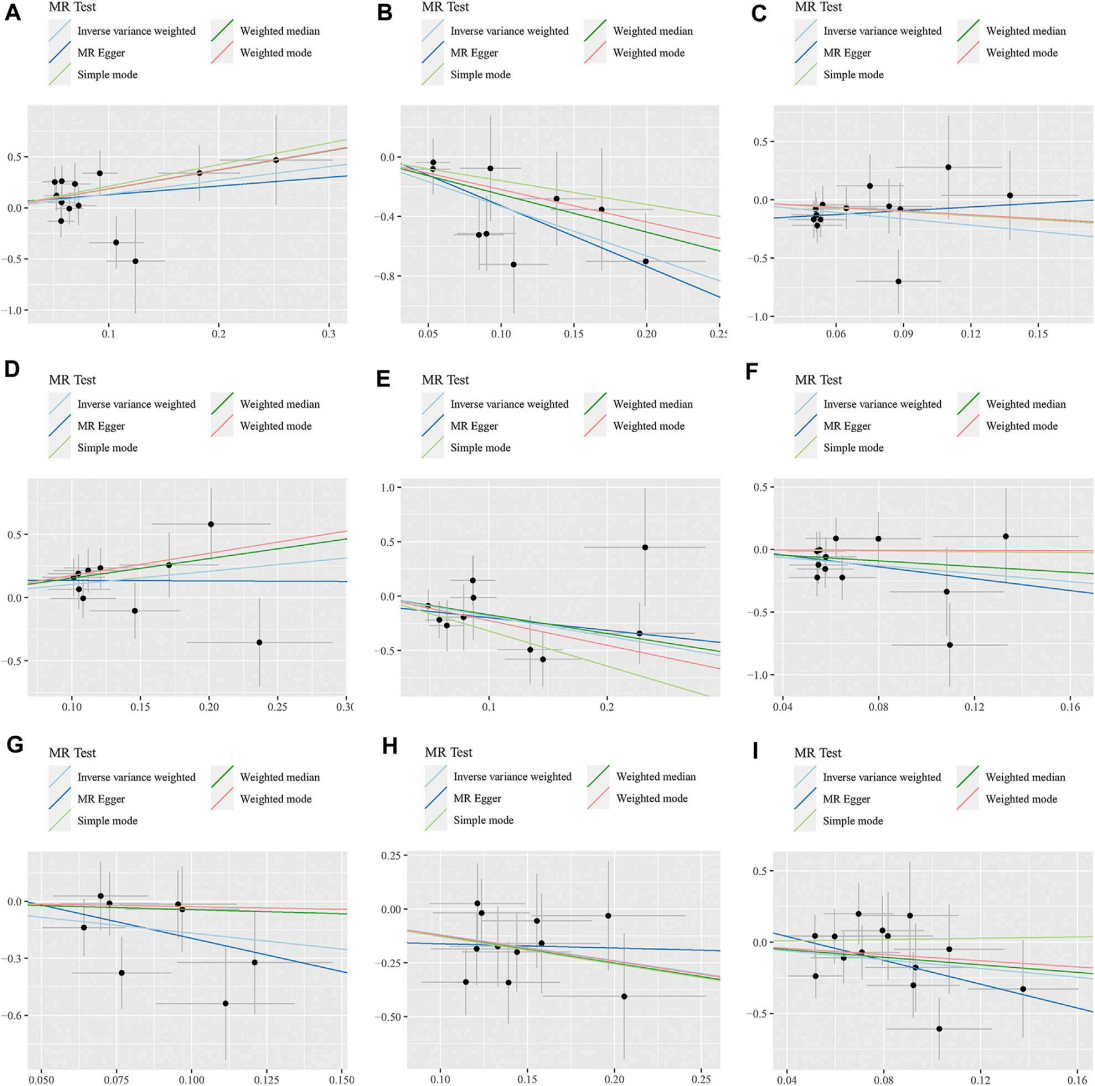
Scatter plots for the causal association between 9 gut microbiota and GBM. **(A)** A. *family.Peptostreptococcaceae;* **(B)** *family.Ruminococcaceae;* **(C)** *genus.Anaerostipes;* **(D)** *genus.Eubacterium brachy group;* **(E)** *genus.Faecalibacterium;* **(F)** *genus.Lachnospiraceae UCG004;* **(G)** *genus.Phascolarctobacterium;* **(H)** *genus.Prevotella7;* **(I)** *genus.Streptococcus.*

The WME method has suggested that *family.Peptostreptococcaceae* (OR: 6.42, 95% CI: 1.09–37.71, *p* = 0.040) and *genus.Eubacterium brachy group* (OR: 4.70, 95% CI: 1.46–15.14, *p* = 0.009) are associated with an increased risk of GBM, while *family.Ruminococcaceae* (OR: 0.08, 95% CI: 0.01–0.79, *p* = 0.031) and *genus.Prevotella7* (OR: 0.28, 95% CI: 0.10–0.84, *p* = 0.023) show a negative correlation with GBM. However, there was no observed association between *genus.Anaerostipes*, *genus.Faecalibacterium*, *genus.Lachnospiraceae UCG004*, *genus.Phascolarctobacterium*, *genus.Streptococcus* and GBM (Figures 3, 4).

**FIGURE 5:**
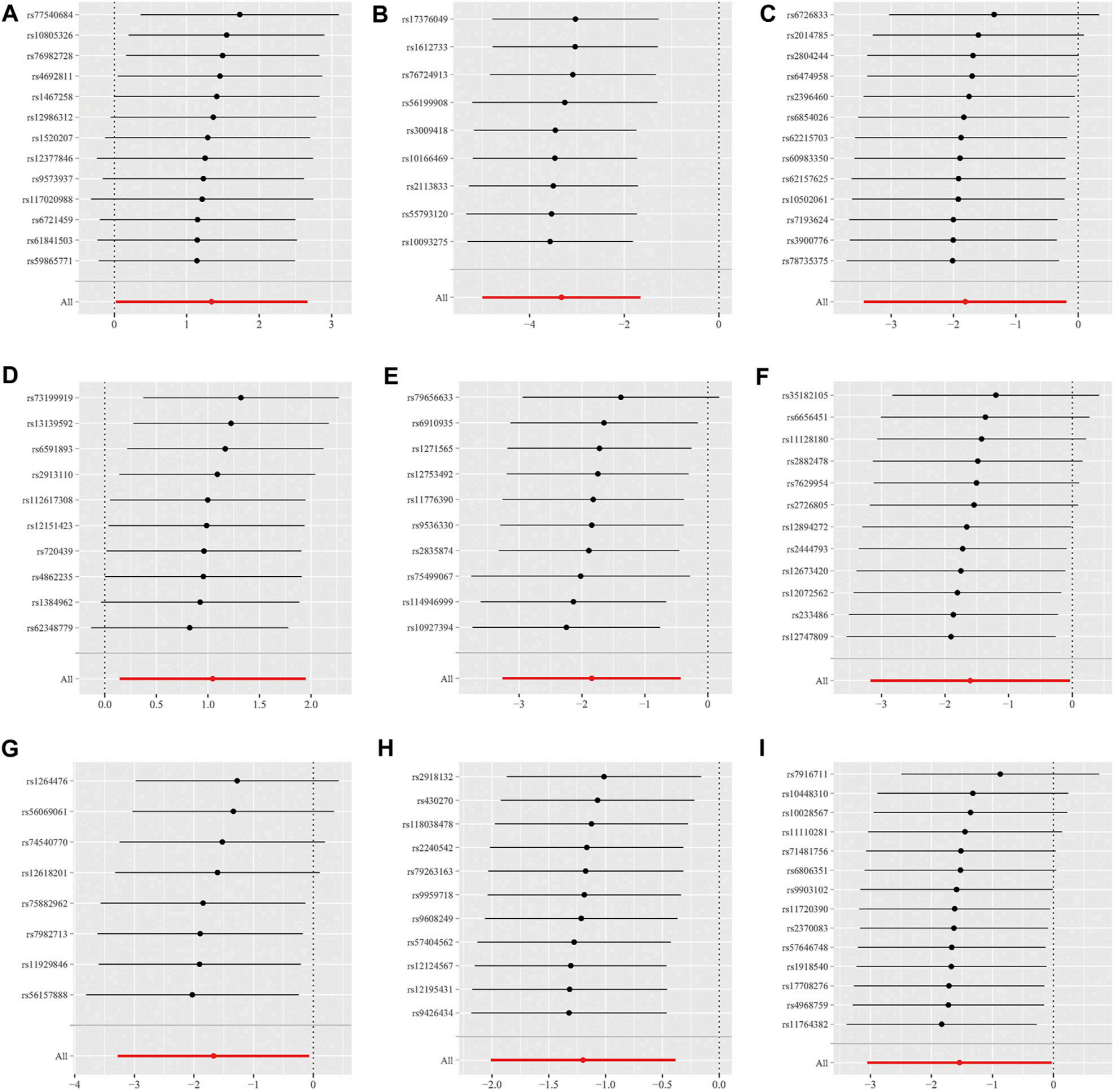
Scatter plots for the causal association between 9 gut microbiota and GBM. **(A)** *family.Peptostreptococcaceae;* **(B)** *family.Ruminococcaceae;* **(C)** *genus.Anaerostipes;* **(D)** *genus.Eubacterium brachy group;* **(E)** *genus.Faecalibacterium;* **(F)** *genus.Lachnospiraceae UCG004;* **(G)** *genus.Phascolarctobacterium;* **(H)** *genus.Prevotella7;* **(I)** *genus.Streptococcus.*

Additionally, the MR-Egger regression intercept did not show evidence of pleiotropy of the gut microbiota with GBM (All intercept *p* > 0.05) (Table 2; Supplementary Table S3). MRPRESSO regression did not identify outliers (All intercept *p* > 0.05).The results of heterogeneity analysis confirmed the accuracy of the findings (Table 2; Supplementary Table S4). Meanwhile, the data’s robustness was further confirmed by the leave-one-out results, which demonstrated a consistent negative association between *family_Ruminococcaceae* and GBM risk (Figure 5; Supplementary Table S5).

**TABLE 2:** Heterogeneity and sensitivity analyses of MR. Abbreviations: MR, Mendelian randomization analysis; SNPs, Number of single nucleotide polymorphism; GBM, Glioblastoma; IVW, Inverse Variance Weighted; MR-PRESSO, Mendelian Randomization Pleiotropy RESidual Sum and Outlier.

Gut microbiota	Outcome	Methods	Q	*P*	Intercept	*P*	MR-PRESSO
***family.Peptostreptococcaceae***	**GBM**						
		IVW	12.152	0.434	0.044	0.735	0.530
		MR-Egger	12.019	0.362			
***family.Ruminococcaceae***	**GBM**						
		IVW	5.115	0.745	0.082	0.670	0.600
		MR-Egger	4.917	0.670			
***genus.Anaerostipes***	**GBM**						
		IVW	8.193	0.770	−0.191	0.338	0.800
		MR-Egger	7.190	0.783			
***genus.Eubacterium brachy group***	**GBM**						
		IVW	8.011	0.533	0.141	0.562	0.850
		MR-Egger	7.645	0.469			
***genus.Faecalibacterium***	**GBM**						
		IVW	7.745	0.560	−0.083	0.586	0.540
		MR-Egger	7.422	0.492			
***genus.Lachnospiraceae UCG004***	**GBM**						
		IVW	8.505	0.668	0.051	0.817	0.720
		MR-Egger	8.448	0.585			
***genus.Phascolarctobacterium***	**GBM**						
		IVW	5.796	0.670	0.207	0.530	0.660
		MR-Egger	5.359	0.616			
***genus.Prevotella7***	**GBM**						
		IVW	5.465	0.858	−0.142	0.683	0.870
		MR-Egger	5.287	0.809			
***genus.Streptococcus***	**GBM**						
		IVW	11.041	0.607	0.207	0.343	0.640
		MR-Egger	10.066	0.610			

### 3.3 Inverse MR analysis

In the reverse MR, GBM was selected as an exposure factor. However, the results of the MR study did not support a causal relationship between GBM and altered gut microbiota (IVW, OR = 1.012, 95% CI: 0.807–1.268, *p* = 0.921) (Supplementary Table S6).

## 4 Discussion

Our study is the first to identify the existence of a direct causal association between gut microbiota and GBM, indicating that an elevated abundance of gut microbiota, such as the *family.Ruminococcaceae*, is associated with a reduced risk of developing GBM. *Ruminococcus* was one of the first gastrointestinal bacteria to be discovered and plays a crucial role in metabolism (Mizrahi et al., 2021). A study on the inflammatory properties of the *family.Ruminococcaceae* found that it produces metabolites in the form of glucomannan polysaccharides, and that these polysaccharides can prime immune system cells (Teng et al., 2022). During the development of GBM, when the BBB is disrupted in the body and circulating immune cells are suppressed in a immunosuppressive environment, gut microbiota such as C. tumefaciens can further enhance the stimulation of immune system cell production. Thus, this bacterium may be a potential protective factor in the development of GBM.

*Genus Faecalibacterium* has been reported as one of the major butyrate producers found in the intestine (Lopez-Siles et al., 2017). *In vitro* studies have demonstrated that butyrate exhibits antitumor effects, such as inhibiting tumor growth by reducing tumor necrosis factor (TNF) secretion in intestinal epithelial cells and inducing differentiation and apoptosis of tumor cells. Butyrate, as a short-chain fatty acid, serves as a histone deacetylase (HDAC) inhibitor, thereby impeding the activity and life cycle of cancer cells (Modoux et al., 2022). Moreover, butyrate, as a short-chain fatty acid and HDAC inhibitor, enhances CPT1A activity to promote induced regulatory T-cell (iTreg) differentiation. iTreg plays a pivotal role in immunosuppression and maintaining immune homeostasis in brain tissue (He et al., 2022). *Genus.Anaerostipes* also belongs to butyrate-producing bacteria and exhibits anti-inflammatory and immunomodulatory functions (Zhang et al., 2016). Within the *genus_LachnospiraceaeUCG004* can reduce tumorigenesis by modulating the function of tumor immunosurveillance (Carasso et al., 2021). However, further studies are needed to explore its potential in terms of GBM risk protection.Therefore, we suggest that these gut microbiota may play a role in GBM development by modulating immunity.

A growing body of evidence underscores the pivotal role of the gut microbiota in tumor therapy, highlighting its key involvement in both local gut immunity and systemic immunity (Park et al., 2022). A robust microbiota employs direct and indirect mechanisms to resist the colonization and invasion of harmful microorganisms, emerging as an integral component of the human defense against external threats. With this in mind, we focused on exploring whether changes in gut flora abundance are linked to the development of GBM as the central theme of this MR. The brain, characterized by a unique immune environment, establishes a crucial link between the gut microbiome and brain tumors through the gut-brain axis. The principal immune privilege in this connection arises from the presence of the blood-brain barrier (BBB), a highly specialized membrane barrier comprised of endothelial cells. The BBB regulates the entry of soluble substances, including antibodies, metabolites, signaling molecules, and immune cells, into the CNS (Obermeier et al., 2013). Experimental studies have elucidated bidirectional communication pathways linking the gut and the brain, encompassing diverse mechanisms such as neural, endocrine, and inflammatory pathways. These pathways are subject to modulation by alterations in gut wall integrity and BBB permeability. Comparable mechanisms are observed between the gut flora and GBM. Notably, when GBM manifests, it disrupts the BBB, facilitating the infiltration of immune cells from the body into the brain parenchyma. Within this specific microenvironment, these immune cells might experience a context where their functionality becomes suppressed (Oberoi et al., 2016; Zhou et al., 2017). This immunosuppression potentially hampers the efficacy of GBM immunotherapy. Hence, there arises a critical consideration: balancing the composition and abundance of gut microbiota could attenuate immunosuppression within the microenvironment surrounding GBM. This modulation may, in turn, potentiate specific therapeutic effects of GBM.

Gut microbiota may regulate astrocyte activity through microbial metabolism that activates the astrocytic aromatic hydrocarbon receptor (AHR). It has been demonstrated that gut commensal microbiota degrade ichthyosine, producing metabolites that reach the CNS and activate the AHR in astrocytes, thereby limiting CNS inflammation (Rothhammer et al., 2018). Aromatic hydrocarbon receptor signaling intricately regulates peripheral T cell differentiation. Additionally, peripheral T cells recruited to the CNS exert control over astrocytic and microglial responses (Rothhammer and Quintana, 2019). Gramarzki et al. reported that aromatic hydrocarbon receptors in GBM cells drive TGF-B expression. Moreover, they highlighted that aromatic hydrocarbon receptor signaling promotes an immunosuppressive microenvironment in GBM (Gramatzki et al., 2009). These findings collectively suggest that gut microbiota may wield a pivotal role in GBM immune evasion by modulating AHR and, consequently, glioma development. Furthermore, they propose the potential of gut microbiota as therapeutic targets for GBM. The microbiota can regulate local and systemic intestinal immunity, particularly in the induction and maturation of immune cells in the nervous system. Gut microbiota dysregulation has been reported to down-regulate granulocyte macrophage colony-stimulating factor (GM-CSF) signal transduction, leading to significant expression of reactive oxygen species (ROS) in activated immature myelocytes, thereby increasing the inhibitory activity of MDSC against T cells (Deh et al., 2019). In addition, dysregulation of the gut microbiota affects the balance between anti-inflammatory Tregs and pro-inflammatory Th17 cells (Chen and Tang, 2021), downregulates Foxp3 expression on tumor cells (Fan et al., 2022), and leads to inhibition of glioma cell growth and apoptosis.

Changes in the gut microbiota composition alter gut immune-brain communication and promote GBM development by creating a tumor-tolerant microenvironment in the CNS (DAlessandro et al., 2020). Recent studies have shown that after the development of GBM, a significant increase in the structure of the bacterial flora is observed, with a significant increase in *Bacteroidetes*, a decrease in the level of *Bacteroidetes* thickeniensis, an increase in the number of *Ackermannia* and *Verrucomicrobia*, and a decrease in the intestinal metabolites propionic, butyric, and acetic acids (Dono et al., 2020). Disruption of the gut microbiota further alters the tumor microenvironment and affects the antitumor efficacy of chemotherapy (Viaud et al., 2013; Daillère et al., 2016). The effects of chemotherapy have been shown to be remarkable in the treatment of tumors. Notably, the microbiota changes differently at different stages after temozolomide treatment. Specifically, there is an increase in the number of *Ackermannia*, *Bifidobacterium*, and *Verrucomicrobium* 7 days after the first temozolomide treatment. Additionally, an increase in the number of *Ackermannia* is observed in patients who responded positively to immunotherapy with PD-1 blockade, suggesting its potential role in mediating the tumor response to immunotherapy (Routy et al., 2018).

The strength of this study lies in the identification of a causal relationship, providing potential gut microbiota candidates for subsequent functional studies. However, several limitations should be considered: (Sung et al., 2021): the MR analysis utilized GWAS data from a European population, necessitating replication in diverse populations; (Ostrom et al., 2023); the study included a limited range of gut microbiota; obtaining GWAS data from additional gut microbiota was crucial for a more comprehensive exploration of their association with GBM; (Omuro and DeAngelis, 2013); while MR is a highly efficient causal analysis method, validating the potential causal link between gut microbiota and GBM requires animal experiments. Finally, (Chen et al., 2021), the causal relationship between gut microbiota and GBM is multifaceted; exploring the etiology and pathogenesis demands a multi-perspective investigation.

## Data Availability

The original contributions presented in the study are included in the article/Supplementary Material, further inquiries can be directed to the corresponding authors.

## Supplementary material

SUPPLEMENTARY TABLE S1 Effect estimates of the associations between 196 gut microbiota and risk of GBM in MR analyses.

SUPPLEMENTARY TABLE S2 The F number and R2 detect the intensity of the IVs between 196 gut microbiota and risk of GBM in MR analyses.

SUPPLEMENTARY TABLE S3 MR-Egger regression analysis between 196 gut microbiota and risk of GBM in MR analyses.

SUPPLEMENTARY TABLE S4 Testing for heterogeneity between 196 gut microbiota and risk of GBM in MR analyses.

SUPPLEMENTARY TABLE S5 Leave one out between 196 gut microbiota and risk of GBM in MR analyses.

SUPPLEMENTARY TABLE S6 Effect estimates of the associations between GBM and risk of gut microbiota in the reverse MR analyses.
